# Maternal Lead Exposure Impairs Offspring Learning and Memory via Decreased GLUT4 Membrane Translocation

**DOI:** 10.3389/fcell.2021.648261

**Published:** 2021-02-25

**Authors:** Zai-Hua Zhao, Ke-Jun Du, Tao Wang, Ji-Ye Wang, Zi-Peng Cao, Xiao-Ming Chen, Han Song, Gang Zheng, Xue-Feng Shen

**Affiliations:** ^1^Department of Occupational and Environmental Health and the Ministry of Education Key Lab of Hazard Assessment and Control in Special Operational Environment, School of Public Health, Fourth Military Medical University, Xi’an, China; ^2^Department of Health Service, Chinese PLA General Hospital, Beijing, China

**Keywords:** lead, learning and memory, hippocampus synaptic plasticity, glucose transporter 4, PI3K-Akt

## Abstract

Lead (Pb) can cause a significant neurotoxicity in both adults and children, leading to the impairment to brain function. Pb exposure plays a key role in the impairment of learning and memory through synaptic neurotoxicity, resulting in the cognitive function. Researches have demonstrated that Pb exposure plays an important role in the etiology and pathogenesis of neurodegenerative diseases, such as Alzheimer’s disease. However, the underlying mechanisms remain unclear. In the current study, a gestational Pb exposure (GLE) rat model was established to investigate the underlying mechanisms of Pb-induced cognitive impairment. We demonstrated that low-level gestational Pb exposure impaired spatial learning and memory as well as hippocampal synaptic plasticity at postnatal day 30 (PND 30) when the blood concentration of Pb had already recovered to normal levels. Pb exposure induced a decrease in hippocampal glucose metabolism by reducing glucose transporter 4 (GLUT4) levels in the cell membrane through the phosphatidylinositol 3 kinase-protein kinase B (PI3K-Akt) pathway. *In vivo* and *in vitro* GLUT4 over-expression increased the membrane translocation of GLUT4 and glucose uptake, and reversed the Pb-induced impairment to synaptic plasticity and cognition. These findings indicate that Pb exposure impairs synaptic plasticity by reducing the level of GLUT4 in the cell membrane as well as glucose uptake via the PI3K-Akt signaling pathway, demonstrating a novel mechanism for Pb exposure-induced neurotoxicity.

## Graphical Abstract

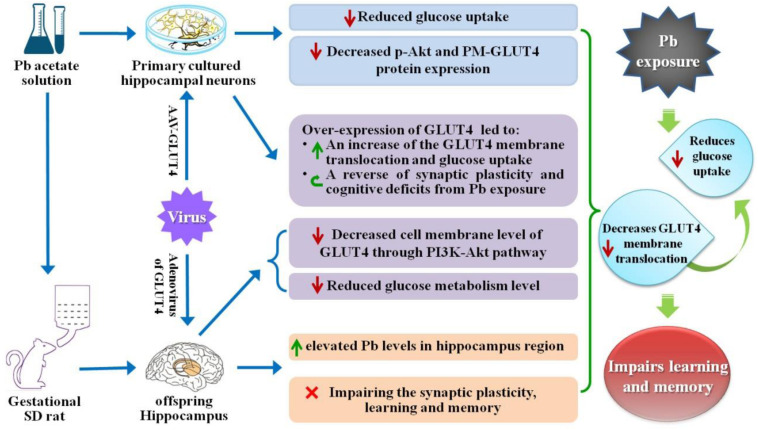

## Highlights

Maternal (pregnancy/gestational) Pb exposure at a low level induces offspring spatial learning and memory deficits via reducing glucose uptake through the down-regulation of GLUT4 membrane translocation.

## Introduction

The developing brain is particularly vulnerable to lead (Pb) exposure, which causes persistent behavioral and emotional disturbances, such as cognitive dysfunction, including deficits in executive functioning, attention, and memory (Mills et al., 2011). Not only that, but as the course of the disease lengthens, the brain damage induced by Pb exposure can eventually develop into dementia (Genuis and Kelln, 2015). Researches have confirmed that chronic Pb exposure is an important risk factor for Alzheimer’s disease related neurodegenerative diseases (Bihaqi and Zawia, 2013). In previous studies, animals exposed to Pb during the perinatal (during gestation through weaning), early postnatal [during postnatal day (PND) 1 through weaning], or late postnatal (during weaning and later) periods were used to evaluate the effects of Pb neurotoxicity on the brain and the underlying mechanisms (Brody et al., 1994). Regardless of the exposure time window, these studies and ours collectively revealed that developmental Pb exposure significantly impairs spatial learning and memory (Hu et al., 2014, 2016; Wang et al., 2016).

As the hippocampus is sensitive to Pb exposure and plays a critical role in spatial learning and memory, which is the basis of cognitive ability (Barha and Galea, 2013), the hippocampus has become a research focus for Pb exposure-induced neurotoxicity (Anderson et al., 2013). Pb exposure during development impairs hippocampus-dependent spatial learning and memory via modifying the levels of *N*-methyl-D-aspartate (NMDA) receptor-dependent brain-derived neurotrophic factors (BDNFs) (Neal et al., 2010), hippocampal long-term potentiation (LTP) (Liu et al., 2012), genomic features (Anderson et al., 2013), and epigenetic regulation by manipulating the expression of DNA methyl-cytosine-binding proteins and methyltransferases (Schneider et al., 2013). Previous animal studies on this topic have enriched our knowledge regarding Pb exposure-induced neurotoxicity. Unfortunately, most of the findings have not been translated to clinical applications. Chelation therapy remains the primary method to treat child Pb poisoning as it helps to lower blood Pb levels while alleviating significant Pb-induced cognitive impairment (Rogan et al., 2001; Bhattacharya et al., 2007). Studies have demonstrated that Pb can accumulate in the blood of pregnant women and subsequently enter the fetus through the placental barrier, eventually leading to fetal mental retardation and long-term adverse effects on offspring learning and memory functions. Moreover, this damage is irreversible, even the damage from low-level Pb exposure to pregnant women (Zartarian et al., 2017; Ettinger and Brown, 2018). Therefore, further clarification of the underlying mechanisms for the impairment of learning and memory induced by environmental Pb exposure is essential.

Glucose is the most important and unique energy substance in the brain and plays a key role in maintaining the structural and functional integrity of neurons in the brain (Im et al., 2019; Yun et al., 2019). It is also reported to be one of the main neuro-modulators that promote brain learning and memory (Smith et al., 2011). Consequently, glucose metabolic disorders always cause cognitive impairments (Kleinridders et al., 2014). Studies have reported that Pb exposure alters the activities of enzymes involved in the glucose metabolic process, which can lead to abnormal glucose metabolism and eventually results in brain energy metabolic disorders (Yun and Hoyer, 2000; Ahmad et al., 2018). Additionally, recent research has shown that Pb exposure causes glucose tolerance and insulin resistance (Mostafalou et al., 2015). Several hypotheses have been proposed for the mechanism by which glucose metabolic disorders cause cognitive impairments. These include the “hippocampal hypothesis” (McNay et al., 2000), which posits that cognitive activity can deplete extracellular glucose in the hippocampus and that exogenous glucose administration reverses the depletion while enhancing task performance. Additionally, the “insulin hypothesis” (de la Monte et al., 2011) suggests that major abnormalities in brains with fetal alcohol spectrum disorder (FASD) are mediated by impairments in insulin/insulin-like growth factor (IGF) signaling. However, the specific mechanism for the learning and memory deficits induced by glucose metabolic disorders remains unclear and needs to be further explored.

Glucose transporters (GLUTs) include more than ten protein families. Among these transporters, GLUT4 is expressed in the central nervous system (CNS) and contributes to synapse formation in the hippocampus (Reagan, 2002). During learning and memory formation, GLUT4 is translocated into neuronal membranes and provides a glucose supply to neurons to maintain the structural and functional integrity of synapses through a process involving the phosphatidylinositol 3 kinase-protein kinase B (PI3K-Akt) signaling pathway (Piroli et al., 2007; Grillo et al., 2009; Chiu and Cline, 2010). However, it remains unclear whether Pb exposure has an impact on GLUTs and whether this influence leads to spacial memory impairment. In this study, a gestational Pb exposure (GLE) rat model was established and the cognition of the rats was assessed. Through morphological analysis (Golgi-Cox staining) and LTP recording in the hippocampal cornu ammonis 1 (CA1) region of the offspring, we investigated the effect of low-level gestational Pb exposure on hippocampal synaptic plasticity and examined how the offspring’s spatial learning and memory functions were affected at PND 30 using Morris water maze (MWM) experiments. Furthermore, the hippocampal glucose metabolism was measured using Positron Emission Tomography-Computed Tomography (PET-CT) scanning and the GLUT4 membrane translocation and expression of hippocampal neurons were measured using immunofluorescence staining and western blot (WB). Finally, we investigated the effects of GLUT4 alterations on hippocampal synaptic plasticity and the offspring’s spatial learning and memory functions by over-expressing GLUT4 *in vivo* and *in vitro*. We found that Pb exposure interfered with the expression and translocation of GLUT4 while reducing the glucose supply in hippocampal neurons, leading to spacial memory deficits.

## Materials and Methods

### Study Design

A low-level GLE rat model was established by feeding Pb solutions to female Sprague-Dawley (SD) rats. The experimental gestational rats were randomly assigned to two groups (control and Pb-exposed). Tissue samples were collected from embryonic rats on day 18∼19 (E18∼19) for primary hippocampal neuronal culture and the offspring rats were used in all other experiments in this study. A previous study showed that there are significant differences between female and male infants and children in the GLE model (Gilbert et al., 2005; Fox et al., 2008); thus, only male offspring rats were used in the current study to reduce bias. For morphological analysis (Golgi-Cox staining), basilar dendrites from CA1 pyramidal neurons in plates 58∼63 were selected for analysis. An investigator blinded to the study conditions performed neuron selection and tracing. Pyramidal neurons were identified based on the unique triangular shape of their soma and their apical extensions toward the apical surface. β-actin was employed as the internal reference protein in all WB tests. The animal protocol and experiments were approved by the Laboratory Animal Welfare and Ethics Committee of the Fourth Military Medical University.

### Animals and GLE Model

In this study, SD rats were used as experimental animals and were subjected to a 12-h light/dark cycle in a 24°C temperature-controlled room. The animals could obtain food and water at will. Based on the research reports by Gilbert et al. (2005) and Fox et al. (2008) as well as our previous study (Zhao et al., 2018), a dose of 109 ppm was chosen to establish the low-level GLE model. Adult female rats raised alone were randomly divided into two groups: the control group received regular water and the Pb-exposed group was exposed to 109 ppm Pb through drinking solutions containing 0.02% Pb acetate (Fisher Scientific, Pittsburgh, PA, United States). This protocol was initiated 2 weeks before mating and continued throughout the gestation period up to PND 10. The weights of the offspring were recorded on PNDs 0, 10, 21, and 30.

### Pb Concentration Analysis

Rats were taken from the control and Pb-exposed groups at PNDs 0, 10, 21, and 30 (*n* = 6, respectively), anesthetized with an intraperitoneal (i.p.) injection of sodium pentobarbital (60 mg/kg), and sacrificed by decapitation. Venous blood and hippocampal tissues digested by organic tissue solvents were used to determine the Pb concentration. The PerkinElmer 600 atomic absorption spectrometer (AAS) (PerkinElmer, United States) was used to analyze the blood samples and a plasmaQuad3 plasma mass spectrograph (VG Elemental, United Kingdom) was used to analyze the tissue samples.

### MWM Tasks

The MWM was used to evaluate the spatial learning and memory of the rats at PND 30 based on the time they took to find an invisible platform. After 5 days of training, the test was performed and the mean time the rats required to reach the platform was recorded. The DigiBehave system (Jiliang Software Company, Shanghai, China) was used to record the routes taken by the rats. The percentage of rats that arrived at the target quadrant within 2 min was calculated. The detailed protocol was described in our previous study (Zhao et al., 2018).

### Golgi-Cox Staining and Morphological Analysis

#### Golgi-Cox Staining

The rats were anesthetized with i.p. injections of 100 mg/kg sodium pentobarbital and cardiac perfusion was performed with 0.9% saline. The rat brains were subsequently extracted for Golgi-Cox staining.

#### Identification of Target Figures

For each animal, five sections from the hippocampal region were selected. Pyramidal neurons were identified based on the unique triangular shape of their soma and the extensions of their apex toward the apical surface. Basilar dendrites from the hippocampal pyramidal neurons in plates 58–63 in the CA1 region were selected for analysis. Note that neuronal selection and tracing were performed by an experimenter who was blinded to the experimental conditions.

#### Neuronal Plasticity Analysis

In order to collect information about the changes in the complexity of the dendritic tree, all basal dendritic segments and the cell body of each neuron were reconstructed using a computer-based neuron-tracking system (Bitplane Imaris, Bitplane AG, Zürich, Switzerland). The spines on a terminal dendrite at the tip of the third order from each selected neuron were counted at a magnification of 1000× to measure the spine density (minimal length 20 μm). The spine structure and spine density of each selected dendrite were assessed using the Bitplane Imaris software.

Please refer to our previous study for the specific procedures used in the above experiment (such as detailed steps for Golgi-Cox staining, observation under a microscope, specific inclusion criteria for the Golgi-impregnated neurons in our study, and spine reconstruction and classification) (Zhao et al., 2018).

### Hippocampal LTP Recordings

#### Slice Preparations

The offspring rats were decapitated at PND 30 after anesthesia (*n* = 6, respectively). The brains were quickly removed and immersed in pre-cooled oxygenated (nearly 0°C, 95% O_2_/5% CO_2_) artificial cerebrospinal fluid (ACSF) containing (in mM): dextrose 10, KCl 5, NaCl 124, NaHCO_3_ 26, NaH_2_PO_4_ 1.25, MgCl_2_ 1.25, and CaCl_2_ 2.5, pH = 7.30∼7.45. Subsequently, the hippocampus of one hemisphere was extracted and sliced (300 μm) using a sliding microtome (Leica VT1000S, Germany). The hippocampal slices were allowed to recover in ACSF at room temperature (RT) for at least 1 h.

#### Electrophysiological Recordings

One slice at a time was transferred into the recording chamber (BSC-HT Med. Sys., United States) where the slice was continuously perfused with 28∼30°C ACSF (95% O_2_/5% CO_2_) at a rate of 1 mL/min. Field excitatory postsynaptic potentials (fEPSPs) were recorded by placing a bipolar stimulating electrode in the Schaffer/commissural fibers while a glass micropipette recording electrode (diameter: 3∼5 μm, resistance: 1∼3 MΩ) was positioned in the dendrites of a CA1 pyramidal cell. The evoking interval for the fEPSPs was 50 s. LTP was induced with a 100 Hz high-frequency stimulation (HFS) for 1 s after recording the baseline responses for 10 min. LTP referred to an increase in the EPSP slope relative to the baseline response (100%) for 40∼45 min after the application of a tetanic stimulus.

### Radioligand Preparation and Positron Emission Tomography/Computed Tomography (PET/CT) Imaging

Fluoro-deoxy-glucose (FDG) scanning was performed in the Small Animal Imaging Core at the Department of Nuclear Medicine, Xijing Hospital. The rats received a tail vein injection of ^18^F-FDG at a dose of 5 μCi/g in saline according to their weights before imaging. After 40 min of uptake while awake, a combination of 5% isoflurane and 95% O_2_ was used to anesthetize the rats. The rats were placed in the dedicated PET-CT camera animal enclosure (nanoScan PET/CT, Mediso) in a head-prone position and high-density PET image reconstruction and CT attenuation correction scanning were performed. The body temperature of the rats was maintained at 37°C via a heating pad. All images were measured with a Derenzo phantom and reconstructed with the Tera-Tomo^TM^ 3D PET engine. In order to quantify the FDG, the regional SUV was quantified with PMOD version 3.801 (PMOD Technologies, Zurich, Switzerland) to obtain the FDG uptake, which was expressed as the SUV.

### Hippocampal Neuron Culture and Virus Transfection

The hippocampal tissue of embryos from normal pregnant SD rats from embryonic day 18∼19 (E18∼19) without Pb exposure was extracted for hippocampal neuron culture and transfection. The hippocampus tissue from E18∼19 of SD rat was extracted in pre-cooled D-Hank solution. Aspirating tissue pieces with as little solution as possible (about 1 mm^3^), and then accutase (Gibco, United States) was added and incubated for enough time at 37°C. After digestion, accutase solution and clasts were removed, Neurobasal (Gibco, United States) media supplemented with B27 and glutamax (GIBCOBRL, United States) was added to suspend the cells. Whereafter, the cells were planted at 2 × 10^5^ cells/mL in 6-well plates precoated by poly-L-lysine. The medium was exchanged every 2 days. In the subsequent experiment, neurons were transfected with the corresponding virus of over-expression GLUT4 at 1 μl (2.5 × 10^9^ ifu/ml) per well for 6-well plates at DIV 6 while cultures were treated with lead acetate (1 μM, Sigma-Aldrich, United States) for 5 days. The details were the same as in our previous study (Zhao et al., 2018).

### Cell Membrane Protein Extraction and Western Blot Analysis

The membrane protein of the hippocampus and primary cultured hippocampal neurons were collected. Equal amounts of protein samples (50 μg) were used for WB analysis as described in our previous study (Zhao et al., 2018). The following were the primary antibodies used in the experiment: GLUT4 (ab654), GLUT1 (ab115730), and GLUT3 (ab41525) (dilution ratio 1:200, Abcam), and β-actin (A5441, dilution ratio 1:1000, Sigma). The peroxidase-conjugated secondary antibodies were F0382 and F9137 (dilution ratio 1:1000, Sigma).

### Immunofluorescence Staining

The rats were anesthetized on PDN 30 with 100 mg/kg sodium pentobarbital (i.p.) and cardiac perfusion was performed with 0.9% saline. Some of the brains in each group were extracted for immunofluorescence staining as described in our previous study (Zhao et al., 2018). The primary antibodies and dilution criterions were as follows: ab654, rabbit anti-GLUT4, 1:200; ab177487, mouse anti-NeuN, 1:400; ab7260, and mouse anti-GFAP, 1:800 (Abcam, United States). The secondary antibodies were as follows: A-21145, Alexa Fluor 594, A-11008, and Alexa Fluor 488 (1:1000, Molecular Probes, Invitrogen). All sections were incubated with Hoechst 33342 (Sigma-Aldrich, St. Louis, MO, United States) for 15 min while avoiding light.

### Glucose Intake Test

Since day DIV 7 to DIV 12 is the primary time period of dendritic spine growth (Zhao et al., 2018), therefore, to establish Pb-exposure model *in vitro*, 1 μM of Pb acetate (Sigma-Aldrich, United States) was added to the cultured hippocampal neurons for 5 days in this period. On DIV 12, the primary culture medium for the hippocampal neurons was replaced with sugar-free neurobasal medium and the neurons were further cultured for 2 h. Afterward, the neurons were cultured with 100 μM 2-(N-(7-Nitrobenz-2-oxa-1,3-diazol-4-yl) amino)-6-deoxy-glucose (2-NBDG) and 1 μg/ml insulin for 30 min. The cells were subsequently collected for flow cytometry after being resuspended in PBS.

### Construction of Neuron-Specific AAV-GLUT4 and Virus Injection

To produce neuron-specific recombination adeno-associated virus (rAAV), the *GLUT4* gene was sub-cloned into plasmids (pAOV-SYN-MCS-EGFP-3FLAG) containing a neuron-specific human synapsin I promoter (hSyn) to generate recombinant plasmids (pAOV-SYN-Slc2a4-P2A-EGFP- 3FLAG) by the company Obio Technology Corp., Ltd (Shanghai, China). AAV-293 cells were then transfected with AAV Rep/Cap expression plasmid, AAV helper plasmid (pAAV Helper), and pAOV-SYN-Slc2a4-P2A-EGFP-3FLAG simultaneously to obtain rAAV (AAV-GLUT4-GFP). Subsequently, viral particles were purified via iodixanol step-gradient ultracentrifugation. The genomic titer (2.5∼3.5 × 10^12^ genomic copies per ml) was determined using quantitative polymerase chain reaction (qPCR). Rats were anesthetized with sodium pentobarbital (60 mg/kg, i.p.) before brain stereo localization injection and placed on a brain stereoscopic positioning injector in a prostrate position to maintain the head level. The bregma and lambda point were identified using a locator and confirmed to be at the same level according to the rat brain map. Specific locations in the hippocampus were also determined (bregma backward 3.96 mm, the midline around 3 mm, bregma down 3.2 mm). A skull drill was used to mark the desired locations on the skull by lightly drilling until the skull cracked, after which the drilling was stopped immediately. According to the set parameters, the needle was slowly inserted at the designated positions and a total amount of 1 μl virus was slowly injected in each site at a speed of 0.05 μl/min. The needle was not moved until the injection was completed and the virus had spread, after which the needle was slowly withdrawn. The micro-injector was moved to a marked position on the other side and the virus was injected in the same manner. After the injection in the bilateral hippocampi was completed, the scalp of the rat was sutured.

### Spinal Analyses of Cultured Neurons

Each experiment was conducted at least three times in this study. Data used for the calculations were presented as the mean ± SEM from a minimum of three independent biological samples. The final results were analyzed with the student’s *T*-test when the number of groups was two, and multiple comparisons were analyzed using one-way analysis of variance (ANOVA) with Dennett’s *post hoc* test. All analyses were performed using the Statistical Package for the Social Sciences (SPSS 20.0) software. *p* < 0.05 was considered to indicate a statistical difference.

### Statistics

Each experiment was carried out at least three times in this study. Data used for the calculations were presented as the mean ± SEM from a minimum of three independent biological samples. The final results were analyzed with the *T*-test when the number of groups was two, and multiple comparisons were analyzed using one-way ANOVA with Dennett’s post-test. All analyses were performed with SPSS 20.0 software. The levels of significance were represented with the following symbols: ^∗^*p* < 0.05 and ^∗∗^*p* < 0.01. *P* < 0.05 was considered to indicate a statistical difference.

## Results

### Low-Level Maternal Pb Exposure Impaired Spatial Learning and Memory in the Offspring

**FIGURE 1:**
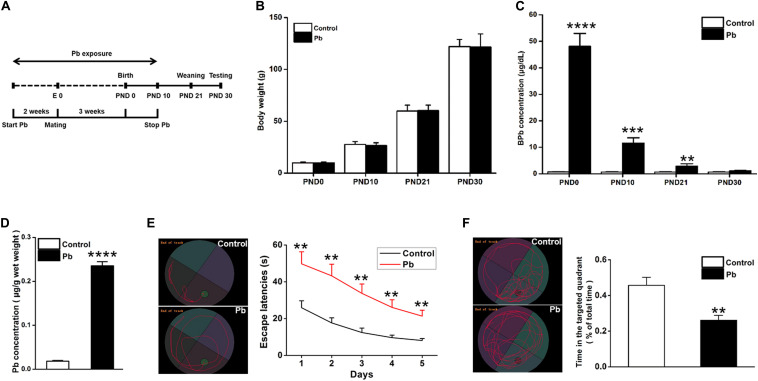
Accumulation of Pb in the hippocampus of PND30 rats and its influence on learning and memory. **(A)** Establishment of Pb exposure model. Female SD rats were given Pb acetate solutions to drink (0 or 0.02%) beginning 2 weeks before mating until PND 10. **(B)** Body weights and **(C)** Blood Pb levels. The concentration of blood Pb in the experimental rats was measured at PND 0, 10, 21, and 30. **(D)** Pb levels in the hippocampus region. **(E,F)** Analysis of MWM results. The MWM tests were only measured at PND30. Data are expressed as mean ± SE, **p < 0.01, ***p < 0.001, ****p < 0.0001; *n* = 6 per group.

Lead (Pb) exposure was initiated 2 weeks before mating, maintained throughout gestation, and terminated 10 days after the progeny were born (Figure 1A). There were no significant differences in the body weights of the offspring at PNDs 0, 10, 21, and 30 between the Pb-exposed and control groups (*p* = 0.9736, *p* = 0.7677, *p* = 0.9188, *p* = 0.9172) (Figure 1B). Blood Pb levels were substantially elevated in the Pb-exposed offspring at PNDs 0, 10, and 21 (*p* < 0.0001, *p* = 0.0001, *p* = 0.0021) (Figure 1C). Though the blood Pb levels in the offspring were not significantly different between the two groups, hippocampal Pb levels were significantly higher in the Pb-exposed offspring at PND 30 (*p* < 0.0001) (Figure 1D). We found that maternal Pb exposure impaired the offspring’s learning and memory abilities, evidenced by longer escape latency during the 5 training days (*p* = 0.009, *p* = 0.0026, *p* = 0.0019, *p* = 0.003, *p* = 0.0015) (Figure 1E) and a significantly shorter amount of time spent in the target quadrant where the platform was originally located (*p* = 0.0037) (Figure 1F) on the testing day compared to those in the control group.

### Low-Level Maternal Pb Exposure Impaired Hippocampal Dendritic Plasticity

**FIGURE 2:**
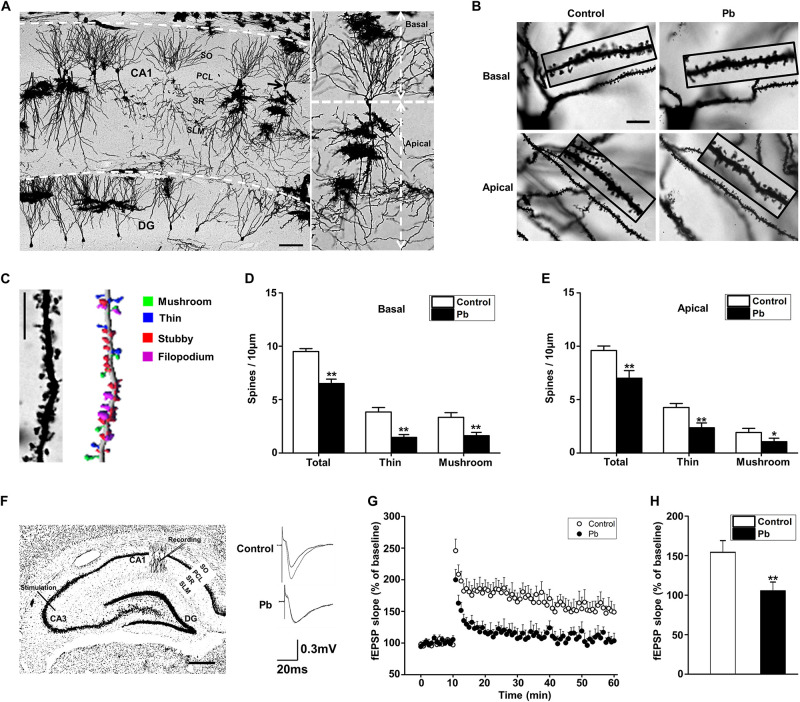
Hippocampal Pb accumulation and impairment of synaptic plasticity in PND30 rats. **(A,B)** Golgi Staining for basal and apical dendritic spines in hippocampal CA1 region. Scale bars = 100 μm in **(A)** and 10 μm in **(B)**. **(C)** Imaris reconstruction of the dendritic spines of pyramidal neurons in CA1 region. Scale bar = 10 μm. **(D,E)** Statistical analysis of the effect of Pb exposure on basal and apical spines. For each group, data are expressed as mean ± SE of spines per 10 μm (16 neurons/8 rats for all groups), *p < 0.05, **p < 0.01. **(F)** LTP induced by high-frequency tetanic stimulation. Simulations were applied to the CA3 regions of the hippocampus and the signal was collected at the CA1 region. Scale bar = 500 μm. **(G,H)** LTP in hippocampal regions CA3-CA1. Abscissa represented the time of tetanic stimulation. *N* = 16 slices per 8 rats in each group. The patch-clamp recordings showed that the fEPSP slope for rat hippocampal LTP in the Pb-exposed group was significantly reduced compared with that in the control group. Data are expressed as mean ± SE, **p < 0.01. SO, stratum oriens; PCL, pyramidal cell layer; SR, stratum radiatum; SL-M, stratum lacunosum-moleculare; DG, dentate gyrus.

Dendritic plasticity is the basis of learning and memory functions. To investigate the underlying mechanisms for the cognitive deficits induced by maternal Pb exposure, we first determined the dendritic plasticity through Golgi Staining and electrophysiology. We reconstructed and analyzed the dendritic spines of pyramidal neurons in the CA1 region using the Imaris software (Figures 2A–C). Pb exposure significantly decreased both the basal (*p* = 0.0018, *p* = 0.0017, *p* = 0.0066) (Figure 2D) and apical (*p* = 0.0021, *p* = 0.0037, *p* = 0.04) (Figure 2E) dendritic spine density of hippocampal CA1 region neurons compared to those in the control group. We found that maternal Pb exposure significantly reduced the fEPSP slope of LTP in the CA1 region (Figure 2F) in the offspring (*p* = 0.0096) (Figures 2G,H). Taken together, maternal Pb exposure impaired hippocampal dendritic plasticity.

### Maternal Pb Exposure Reduced Glucose Metabolism in the Offspring Hippocampus

**FIGURE 3:**
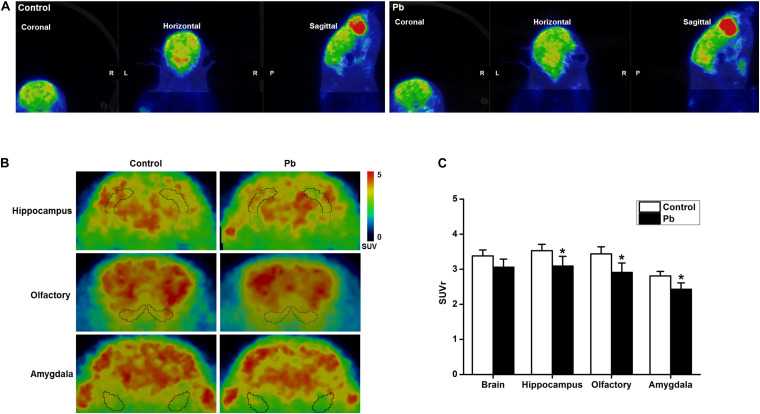
Effect of low-level gestational Pb exposure on glucose uptake in rat brain. **(A)** Representative images of experimental rat brains. **(B)** Representative images of glucose uptake in different brain areas ([18F]-FDG microPET/CT) in the experimental rats at the last scanning point. **(C)** Statistical analysis of PET-CT scanning data. PET-CT results indicated that maternal Pb exposure at a low level significantly reduced the level of glucose metabolism in the rat hippocampus. *p < 0.05, *n* = 6 per group.

Positron Emission Tomography-Computed Tomography PET-CT scanning was used to analyze the effects of maternal Pb exposure on glucose metabolism in the offspring brain (Figure 3A). The glucose uptake profiles in the offspring hippocampus, olfactory bulb, and amygdala are shown in Figure 3B. The results showed that the levels of glucose metabolism in the hippocampus, amygdala, and olfactory bulb in the Pb-exposed group were significantly reduced compared with those in the control group. The standard uptake value (SUV) for the hippocampus were 3.52 ± 0.12 in the control group and 3.06 ± 0.16 in the Pb-exposed group (*p* = 0.0418). The corresponding values for the olfactory bulb were 3.49 ± 0.14 and 2.93 ± 0.21 (*p* = 0.0487) and the values for the amygdala were 2.84 ± 0.09 and 2.46 ± 0.14 (*p* = 0.048), respectively (Figure 3C, *n* = 6).

### Maternal Pb Exposure Led to Alterations in Rat Hippocampal Neuron GLUT4 Levels and Membrane Translocation

**FIGURE 4:**
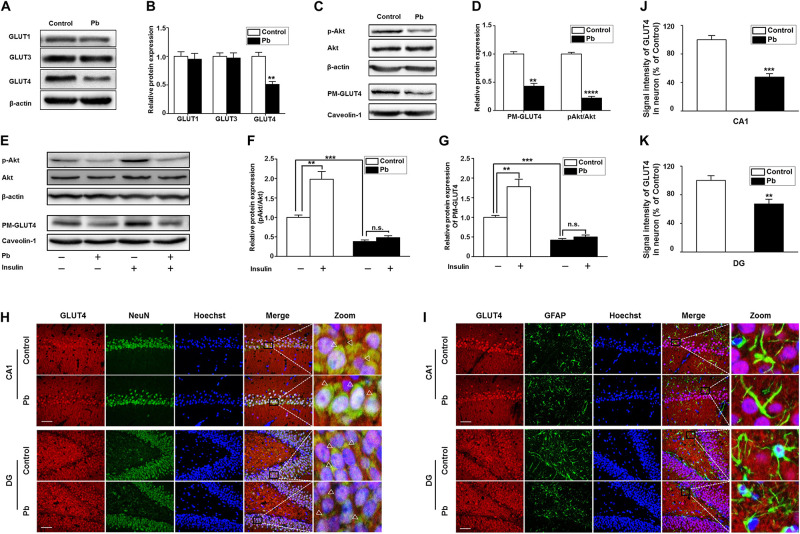
Effects of Pb exposure on GLUT4 expression and membrane translocation in rat hippocampus. **(A–D)** Effects of Pb exposure on the expression of GLUTs, PM-GLUT4, and p-Akt. There were no remarkable changes in GLUT3 and GLUT1 expression in Pb-exposed rat hippocampus, but the GLUT4 expression was significantly reduced. The expression levels of PM-GLUT4 and p-Akt were significantly reduced. Among them, caveolin-1 served as the loading control of PM-GLUT4, and β-actin for other proteins. **(E–G)** Effects of insulin on PM-GLUT4 and p-Akt expression levels. Insulin stimulation significantly increased the expression of p-Akt in rat hippocampus but did not reverse the decrease of p-Akt expression in the Pb-exposed group. *N* = 6 per group, **p < 0.01, ***p < 0.001, ****p < 0.0001. vs. control. The band intensity was statistically analyzed based on the percentage relative to the control. **(H,I)** Immunofluorescence staining. **(J,K)** Statistical results of signal intensity of GLUT4 expression in neuron in CA1 and DG regions of the hippocampus in rats. The results showed that GLUT4 and NeuN were significantly double-labeled in the CA1 and DG regions of the hippocampus in rats, and double-labeling in the Pb-exposed group was significantly reduced compared to the control group levels. Immunofluorescence staining indicated that GLUT4 and GFAP were not double-labeled in the hippocampal CA1 and DG regions of rats. Scale bars = 50 μm. Data were represented as mean ± SEM and counted based on data from a minimum of three independent experiments, **p < 0.01, ***p < 0.001.

Glucose plays a vital role in maintaining the integrity of synaptic structures and functions (McNay and Gold, 2002; Im et al., 2019; Yun et al., 2019). The disruption of brain glucose metabolism can impair neuronal synaptic plasticity, which in turn affects brain function. We found that the protein levels of GLUT1 and GLUT3 in the offspring hippocampus were not significantly different between the two groups (*p* = 0.5869, *p* = 0.8793) (Figures 4A,B); however, the GLUT4 protein level was significantly lower in the Pb-exposed group (*p* = 0.0012) (Figure 4B). The membrane protein levels of GLUT4 in the hippocampal neurons of the Pb-exposed offspring were significantly reduced compared with those in the control group (*p* = 0.0014) (Figures 4C,D), and the phosphorylated level of Akt (p-Akt) was lower (*p* < 0.0001) (Figures 4C,D). The results showed that exogenous insulin alone was capable of inducing a significant increase in Akt phosphorylation and GLUT4 translocation (*p* = 0.0016, *p* = 0.0058) in the normal group (Figures 4E–G). However, in the Pb exposure group, insulin treatment neither reversed the phosphorylation level of Akt nor changed the level of GLUT4 membrane translocation (*p* = 0.1357, *p* = 0.1456) (Figures 4E–G).

GLUT4 co-localized with NeuN (Figure 4H) but not glial fibrillary acidic protein (GFAP) (Figure 4I) in the hippocampal CA1 and dentate gyrus (DG) regions, which means that the GLUT4 proteins express in neurons of hippocampus, not astrocytes during brain development in rat offspring. We also found that compared with the control, the immunoreactivity of GLUT4 significantly decreased in the CA1 and DG regions (*p* = 0.003, *p* = 0.0098) (Figures 4J,K). These data suggest that gestational Pb exposure causes a decrease in the total and membrane levels of GLUT4 in hippocampal neurons.

### Over-Expression of GLUT4 *in vitro* Increased Glucose Uptake and Reversed Synaptic Plasticity in the Pb-Exposed Group

**FIGURE 5:**
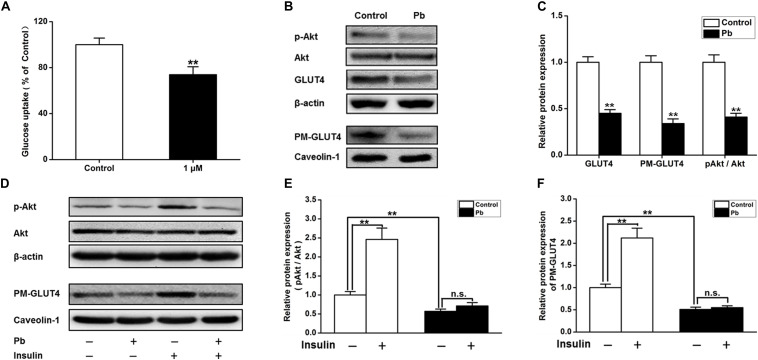
Effects on the glucose uptake and GLUT4, PM-GLUT4, and p-Akt expression in primary hippocampal neurons in the Pb-exposed rats. **(A)** Effect of Pb exposure on the glucose uptake. Using 2-NBDG combined with flow cytometry, glucose uptake in primary cultured rat hippocampal neurons was analyzed. Flow cytometry results showed that glucose uptake was significantly reduced in primary cultured hippocampal neurons after treatment with 1 μmol/L Pb acetate compared with the levels in the control group. **(B,C)** Effect of Pb exposure on expression of glycometabolism-related proteins in the brain. The expression levels of GLUT4, PM-GLUT4, and pAkt proteins in primary cultured hippocampal neurons were dramatically reduced in the Pb-exposed rats. **(D–F)** p-Akt and PM-GLUT4 expression after insulin stimulation in rat primary cultured hippocampal neurons. Insulin stimulation led to the significant increase of p-Akt and PM-GLUT4 protein levels in the control rat hippocampus, but did not reverse the decreased levels in Pb-exposed rats. Among them, caveolin-1 served as the loading control of PM-GLUT4, and β-actin for other proteins. **p < 0.01, *n* = 6 per group. Statistical analysis of the band intensity measured as a percentage of the control.

The glucose uptake of neurons was significantly reduced after Pb exposure (*p* = 0.0092) (Figure 5A). Compared with the control group, the expression levels of total and membrane GLUT4 proteins as well as p-Akt proteins in the Pb-exposed group were significantly decreased (*p* = 0.0012, *p* = 0.0011, *p* = 0.0015) (Figures 5B,C). Exogenous insulin was administered to primary hippocampal neurons and the insulin signaling pathway was assessed to determine the effect on the membrane translocation of GLUT4 (Figure 5D). The results showed that the p-Akt levels and membrane translocation of GLUT4 were significantly increased after insulin treatment alone (*p* = 0.0016, *p* = 0.0022). However, insulin treatment in the Pb-exposed group did not reverse the reduced levels of p-Akt and plasma membrane (PM)-GLUT4 proteins (*p* = 0.1245, *p* = 0.3444) (Figures 5E,F).

**FIGURE 6:**
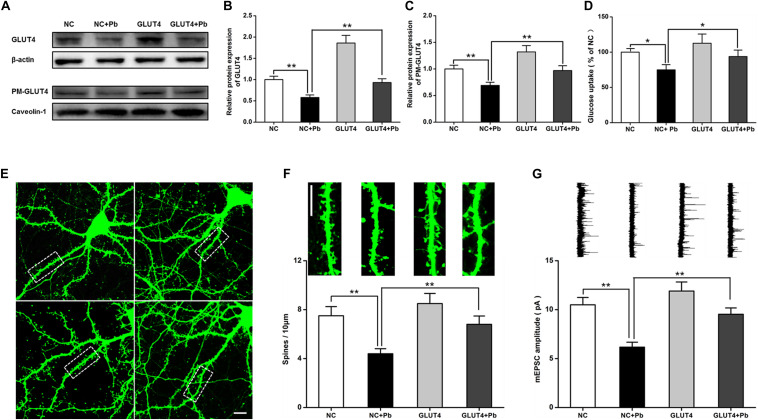
Effects of over-expression of GLUT4 on glucose uptake and synaptic plasticity of primary hippocampal neurons in the Pb-exposed rats. **(A–C)** Expression changes in GLUT4 and PM-GLUT4 in rat hippocampal neurons after AAV-GLUT4 transfection. GLUT4 and PM-GLUT4 expression levels returned to normal in the rat primary cultured hippocampal neurons of the Pb-exposed group after AAV-GLUT4 transfection. Among them, caveolin-1 served as the loading control of PM-GLUT4, and β-actin for GLUT4. **(D)** Effect of GLUT4 over-expression on glucose uptake in rat hippocampal neurons. Over-expression of GLUT4 recovered the glucose uptake levels in the primary hippocampal neurons from the Pb-exposed group. **(E–G)** Effects of GLUT4 over-expression on cell morphology and LTP in Pb-exposed rat hippocampal neurons. After lentivirus infection, the morphology (dendritic spines) of rat primary cultured hippocampal neurons was observed via laser confocal microscopy. Over-expression of GLUT4 reversed the dendritic spine density and LTP lesions in Pb-exposed rats. Scale bar = 10 μm. *p < 0.05, **p < 0.01, vs. control, *n* = 6 per group.

After GLUT4 over-expression (Figures 6A,B), the transmembrane levels of GLUT4 in the primary hippocampal neurons of the Pb-exposed group were significantly increased (*p* = 0.0091, *p* = 0.0099) (Figure 6C), whereas glucose uptake by hippocampal neurons in the Pb-exposed group was significantly decreased (*p* = 0.0116, *p* = 0.0499) (Figure 6D). The density of dendritic spines in the hippocampal neurons of the Pb-exposed group was significantly decreased compared to that of the control group and this trend was significantly reversed after the over-expression of GLUT4 (*p* = 0.0037, *p* = 0.0071) (Figures 6E,F). In addition, the amplitude of miniature excitatory postsynaptic currents (mEPSCs) from hippocampal neurons in the Pb-exposed group was significantly lower. The over-expression of GLUT4 significantly increased the amplitude of mEPSCs in the Pb-exposed group (*p* = 0.001, *p* = 0.0035) (Figure 6G).

### Over-Expression of GLUT4 *in vivo* Reversed Cognitive Impairments Induced by Gestational Pb Exposure by Increasing GLUT4 Membrane Translocation and Glucose Uptake

**FIGURE 7:**
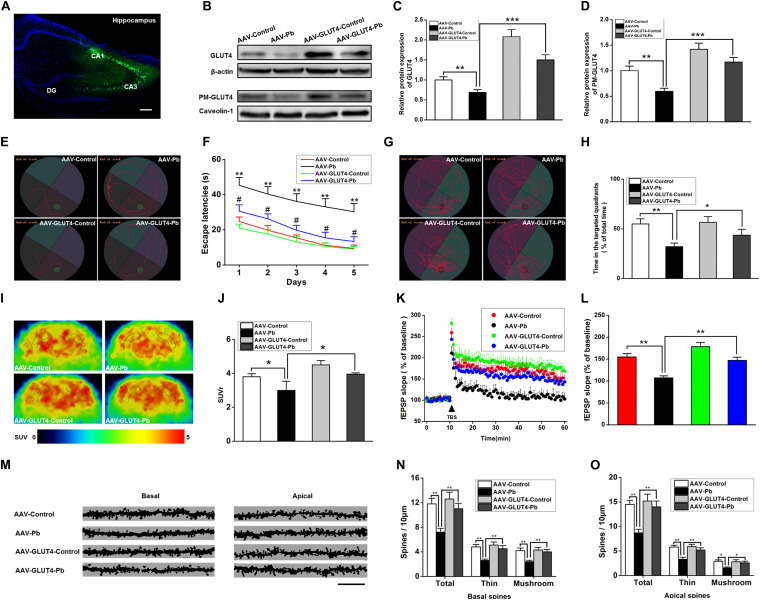
Effects of GLUT4 over-expression in Pb-exposed rat hippocampus on the glucose uptake, synaptic plasticity, as well as learning and memory function. **(A–D)** Over-expression of GLUT4 in rat hippocampus and analysis of its effect on glycometabolism-related proteins. GLUT4 over-expression was induced via stereotactic injection of adenovirus. GLUT4 over-expression reversed the decreased PM-GLUT4 in Pb-exposed rat hippocampus. **(E–H)** Analysis of rat positioning, navigation ability, and space exploration trajectory. Over-expression of GLUT4 in Pb-exposed rat hippocampus reversed the arrival latency and the target quadrant retention time of model rats in MWM tests; *p < 0.05, **p < 0.01; # *p* < 0.05 vs. AAV-Pb. **(I,J)** PET-CT scanning and analysis of glucose metabolism in rat brain. The glucose metabolism level in Pb-exposed rat hippocampus was reversed after the over-expression of GLUT4. **(K,L)** Effects of Pb exposure on LTP induction in rat hippocampus. After over-expression of GLUT4 in rat hippocampus, the slope of fEPSP in Pb-exposed rat hippocampus was reversed. **(M–O)** Effect of Pb exposure on dendritic spines of hippocampal pyramidal neurons. Over-expression of GLUT4 increased the spinal density of basal and apical dendrites in pyramidal neurons as well as the number of thin and mushroom-shaped dendritic spines in the Pb-exposed rat hippocampus. Scale bar = 10 μm. *p < 0.05, **p < 0.01, ***p < 0.001, vs. control, *n* = 6 per group.

To investigate whether GLUT4 over-expression attenuates the cognitive deficits caused by Pb exposure, GLUT4 transfected with adeno-associated virus (AAV) and tagged with green fluorescent protein (GFP) (AAV-GLUT4-GFP) was injected into the hippocampal CA1 region. The effects of the virus transfection were demonstrated by a strong positive GFP fluorescence (Figure 7A) and the WB results (Figure 7B). The levels of GLUT4 and PM-GLUT4 proteins in the hippocampus increased after AAV-GLUT4 injection (Figures 7C,D). After receiving the same amount of Pb exposure, the levels of GLUT4 and PM-GLUT4 proteins in the AAV-GLUT4-Pb group were lower compared with the levels in the control group (AAV-GLUT4-control) (*p* = 0.0082, *p* = 0.0026), but considerably higher than those in the AAV-Pb group (*p* = 0.0004) (Figures 7C,D). Using the MWM test, we found that the over-expression of GLUT4 significantly ameliorated the cognitive deficits induced by Pb exposure, evidenced by a decrease in the escape latency (Day 1: *p* = 0.0024, *p* = 0.0281; Day 2: *p* = 0.0024, *p* = 0.0214; Day 3: *p* = 0.0013, *p* = 0.0105; Day 4: *p* = 0.0011, *p* = 0.0102; Day 5: *p* = 0.0019, *p* = 0.0103; vs. AAV-Pb) (Figures 7E,F) and a longer period of time spent in the target quadrant (*p* = 0.005, *p* = 0.0428) (Figures 7G,H). GLUT4 over-expression also significantly reversed the impaired hippocampal glucose metabolism in the Pb-exposed group, as determined using PET-CT (*p* = 0.0487, *p* = 0.0188) (Figures 7I,J). AAV-GLUT4 significantly attenuated the inhibitory effect of Pb exposure on hippocampal LTP, indicated by a slope of 108.18 ± 4.96 for fEPSPs in the AAV-Pb group and 146.67 ± 7.83 in the AAV-GLUT4-Pb group (*p* = 0.002) (Figures 7K,L). Changes in dendritic plasticity in rat hippocampal neurons after GLUT4 over-expression were observed (Figure 7M, *n* = 6). When GLUT4 was overexpressed, the total numbers of basal and apical dendrites in the CA1 region of the Pb-exposed rats were significantly higher (*p* = 0.0055), as were the densities of thin- and mushroom-shaped spines (*p* = 0.007, *p* = 0.0027) (Figures 7N,O).

## Discussion

Lead (Pb) is a widely distributed environmental heavy metal poison that is a serious health hazard to humans, especially to brain development in children (Olympio et al., 2009; Senut et al., 2012; Hara et al., 2015). However, the mechanism through which Pb damages the developing brain and leads to learning and memory impairments remains unclear (Ettinger and Brown, 2018). According to our experimental results, although the blood Pb level recovered to normal levels in the Pb-exposed offspring at PND 30, the Pb level in the hippocampal tissue from the Pb-exposed group remained substantially higher. Low-level maternal Pb exposure impaired the developing brain, evidenced by increased escape latency and less time spent in the target quadrant. In other words, once the impairment to the developing brain induced by Pb exposure has occurred, it is irreversible and will not be ameliorated even if the blood Pb levels return to normal.

Dendritic plasticity is the basis of learning and memory functions. The density and morphological changes of dendrites have significant effects on excitatory synaptic transmission and are closely related to learning and memory functions (Dumitriu et al., 2010; Haas et al., 2013). Any event that affects the structural foundation (pyramidal cell dendritic spine) of dendrites can eventually lead to impaired LTP as well as cognition. Among these events, hippocampal neuronal (Bott et al., 2013) plasticites are one of the most important factors that can alter the hippocampal LTP. Dendritic spines are classified into the following four categories according to their structural and functional characteristics: mushroom, thin, stubby, and filopodium (Larance et al., 2008). Mushroom and thin dendritic spines are closely associated with learning and memory ability (Fiala et al., 1998; Zhao et al., 2018). In this study, we discovered that the spine densities of the basal and apical dendrites were significantly reduced in the hippocampus of GLE offspring, especially the thin and mushroom spines. Combined with the decreased LTP, maternal Pb exposure impaired the learning and memory abilities of the offspring through synaptic plasticity alterations.

Learning and memory are complex neurophysiological processes that require a large exogenous glucose supply since glucose cannot be synthesized or stored in neurons (Messier, 1997; Hughes and Neeson, 2003; Chen and Zhong, 2013) and must be transported into the neurons by glucose transporters. GLUT4 is expressed in brain regions associated with cognitive behavior, including the hippocampus, amygdala, olfactory bulb, cerebral cortex, and cerebellum (Nagamatsu et al., 1993; Leloup et al., 1996; Ngarmukos et al., 2001). Insulin-mediated GLUT4 membrane translocation is responsible for the hippocampal glucose supply during learning and memory (McNay and Gold, 2002; Grillo et al., 2009; Ren et al., 2019). In this study, Pb exposure had no significant effect on blood glucose levels in rats, but GLUT4 expression was significantly reduced in the hippocampal neuronal membrane proteins after Pb exposure. This suggests that Pb exposure affects the energy supply in the hippocampus-dependent learning and memory process by reducing the membrane translocation of GLUT4. We also found that Pb exposure significantly decreased GLUT4 expression. To further confirm the key role of GLUT4 in learning and memory deficits induced by Pb exposure, we overexpressed GLUT4 using the AAV-GLUT4 virus and evaluated synapse plasticity and spatial memory. GLUT4 over-expression increased the amount of GLUT4 translocated across the membrane as well as glucose uptake in the hippocampus, as determined with PET-CT. These data suggested that GLUT4 is a key molecule that mediates glucose uptake in hippocampal neurons and is also an important toxic target protein for Pb exposure to inhibit neuronal glucose uptake. Over-expression of GLUT4 in the hippocampus also ameliorated learning and memory impairments and the inhibitory effect on hippocampal LTP, as well as the damage to neuronal dendritic spines caused by Pb exposure.

In conclusion, the cognitive defects induced by maternal Pb exposure can persist beyond PND 30 and are not reversed even after the blood Pb concentration returns to normal levels. In other words, this impairment remains in the offspring’s brain throughout the developmental stage. Abnormal glucose metabolism plays a role in the spatial learning and memory impairments induced by maternal Pb exposure during the developmental stage; however, the underlying mechanisms were unclear till now. In this study, we found that Pb exposure during pregnancy irreversibly damaged the developing nervous system, especially the hippocampal synaptic plasticity. The exposure caused learning and memory impairments in the offspring by reducing glucose uptake through a decrease in GLUT4 protein expression and membrane translocation in the hippocampus. Pb exposure inhibited the PI3K-Akt signaling pathway, which is critical for the membrane translocation of GLUT4, both *in vivo* and *in vitro*. Over-expression of GLUT4 ameliorated the synaptic plasticity impairments and cognitive deficits induced by gestational Pb exposure via increasing the GLUT4 membrane translocation and glucose uptake *in vivo* and *in vitro*. These findings indicate that Pb exposure causes memory deterioration through PI3K-Akt-induced suppression of GLUT4 membrane translocation, revealing a novel mechanism for Pb exposure-induced synapse and memory impairments. This research may provide a new therapeutic target for brain development impairments in maternal Pb-exposed offspring.

There are some shortcomings in our investigation. For example, a previous study showed that Pb exposure during pregnancy causes more serious damage to boys than girls (Khanna, 2015), but we only focused on the male offspring. Therefore, the mechanism for Pb-related synapse and memory impairments induced by Pb exposure, which causes memory deterioration through PI3K-Akt-induced suppression of GLUT4 membrane translocation, and its underlying molecular mechanism both require further verification in other populations. Moreover, the neuroligin 1 (NLGN1), which is one of the four subtypes of the postsynaptic neuroligins, is one of the most characteristic synaptic cell adhesion molecules. And we had confirmed that low-level gestational Pb exposure also down-regulated NLGN1 expression and dendritic spine density in hippocampal CA1 pyramidal neurons, leading to impairment of learning and memory, and overexpression of NLGN1 rescued the Pb-induced reduction of spine density in hippocampal neurons in our previous study. These results indicate that low-level gestational Pb exposure seems to have the same impact on NLGN1 and GLUT4, and the protein NLGN1 and GLUT4 play similar roles in synaptic plasticity as well as learning and memory function. But whether there would be some connections between these two findings, and whether there are some signaling pathways or regulatory mechanisms, are still unexplained in the present study. However, it will be an important direction for our future research. The findings will contribute to further translation of the theoretical research into clinical applications, which is also the focus of our future work.

## Data Availability Statement

The original contributions presented in the study are included in the article/supplementary material, further inquiries can be directed to the corresponding authors.

## Ethics Statement

The animal study was reviewed and approved by the Laboratory Animal Welfare and Ethics Committee of the Fourth Military Medical University.

## Author Contributions

Z-HZ and K-JD wrote the manuscript. TW and J-YW developed the model and performed the molecular biology experiments. Z-PC participated in the *in vivo* experiments. X-MC and HS analyzed the data. GZ and X-FS designed and supervised the experiments. All authors read and approved the final manuscript.

## Conflict of Interest

The authors declare that the research was conducted in the absence of any commercial or financial relationships that could be construed as a potential conflict of interest.
